# H-ABC tubulinopathy revealed by label-free second harmonic generation microscopy

**DOI:** 10.1038/s41598-022-18370-x

**Published:** 2022-08-24

**Authors:** Milvia Alata, Valeria Piazza, Carla Jaramillo-Restrepo, Jose R. Eguibar, Carmen Cortes, Victor H. Hernandez

**Affiliations:** 1 Centro de Investigaciones en Optica A.C., Loma del Bosque 115, Colonia Lomas del Campestre, 37150 Leon, Guanajuato, Mexico; 2 Institute of Physiology, Benemérita Universidad Autónoma de Puebla, 14 sur No. 6301, Colonia San Manuel, 72570 Puebla, Mexico; 3 Research Office, Vicerrectory of Research and Postgraduate Studies, Benemérita Universidad Autónoma de Puebla, 14 sur No. 6301, Colonia San Manuel, 72570 Puebla, Mexico; 4 Division of Sciences and Engineering, University of Guanajuato, Loma del Bosque 103, Colonia Lomas del Campestre, 37150 Leon, Guanajuato, Mexico

**Keywords:** Optical imaging, Demyelinating diseases, White matter disease

## Abstract

Hypomyelination with atrophy of the basal ganglia and cerebellum is a recently described tubulinopathy caused by a mutation in the tubulin beta 4a isoform, expressed in oligodendrocytes. The *taiep* rat is the only spontaneous tubulin beta 4a mutant available for the study of this pathology. We aimed to identify the effects of the tubulin mutation on freshly collected, unstained samples of the central white matter of *taiep* rats using second harmonic generation microscopy. Cytoskeletal differences between the central white matter of *taiep* rats and control animals were found. Nonlinear emissions from the processes and somata of oligodendrocytes in tubulin beta 4a mutant rats were consistently detected, in the shape of elongated structures and cell-like bodies, which were never detected in the controls. This signal represents the second harmonic trademark of the disease. The tissue was also fluorescently labeled and analyzed to corroborate the origin of the nonlinear signal. Besides enabling the description of structural and molecular aspects of H-ABC, our data open the door to the diagnostic use of nonlinear optics in the study of neurodegenerative diseases, with the additional advantage of a label-free approach that preserves tissue morphology and vitality.

## Introduction

Tubulinopathies are neurodegenerative diseases generated by mutations in genes that codify for tubulins. In particular, mutations in tubulin beta 4a (TUBB4A) have been related to hypomyelination with atrophy of basal ganglia and cerebellum (H-ABC)^1,2^. Magnetic Resonance Imaging (MRI) features of the syndrome include central hypomyelination and atrophy of the basal ganglia, cerebellum and the corpus callosum^3^. Clinical signs of H-ABC patients encompass extrapyramidal movements, spasticity, ataxia, sensory impairment, and mental disability^1,4–6^.

We have shown that the *taiep*, a spontaneous mutant rat^7^ is the animal model for the study of tubulinopathies^3^. It suffers from hypomyelination at birth and progressive demyelination of the central nervous system^3,7–9^. The radiological features of the murine TUBB4A mutant closely match those of humans suffering from H-ABC^3^. MRIs of *taiep* rats show a progressive thinning of the corpus callosum and hyperintensities in major white matter structures in T2 weighted images, which are related to the loss of myelin in those regions^10^. The demyelination has been related to the accumulation of microtubules (MTs) within oligodendrocytes of *taiep* rats, in the soma, and in the processes^9,11^, a similar accumulation has also been described in an H-ABC patient bearing a different TUBB4A mutation^8^. All of these observations come from either MRI, which has low resolution compared to microscopy, or from electron and optical micrographs of fixed tissue sections. To uncover the cellular features of this complex disease, a method that preserves the sample in a minimally altered condition is preferred, due, among many other things, to the sensibility of tubulin ultrastructure, the mutant protein causing the myelin disorder, to fixing agents^12,13^. For the visualization of native tissue in its physiological context, applications of nonlinear optical phenomena, like second harmonic generation (SHG) microscopy are available, adding also the advantage of label-free emissions for prolonged observation and dynamic studies^14^.

To study tubulinopathies in the central nervous system (CNS), SHG microscopy has the particular advantage that it is able to image unlabeled microtubules. SHG is a second-order nonlinear optical phenomenon that occurs exclusively in non-centrosymmetric media with a large molecular hyperpolarizability^15^. Collagen, muscular myosin, and tubulin are the three non-centrosymmetric biological materials known so far, that form highly ordered arrays, which emit second harmonic signals^16^. When tubulin interacts with an intense short laser pulse, it scatters frequency-doubled photons, which can be exploited for label-free imaging. The only SHG emitters in the central nervous system are collagen and microtubules^17^. Collagen is abundant in the meninges^18^, vessel walls and the choroid plexus^19^, while microtubules, despite being ubiquitous, are highly organized and locally concentrated in the axons^20^. The second harmonic signal intensity from microtubules depends on their direction (parallel or antiparallel), organization and abundance^13,21^. As SHG is a coherent process, the signals from parallel MTs sum up, while signals from antiparallel MTs interfere destructively. It is estimated that a detectable second harmonic signal arises from bundles of about 50, predominantly parallel microtubules^21^.

**Figure 1:**
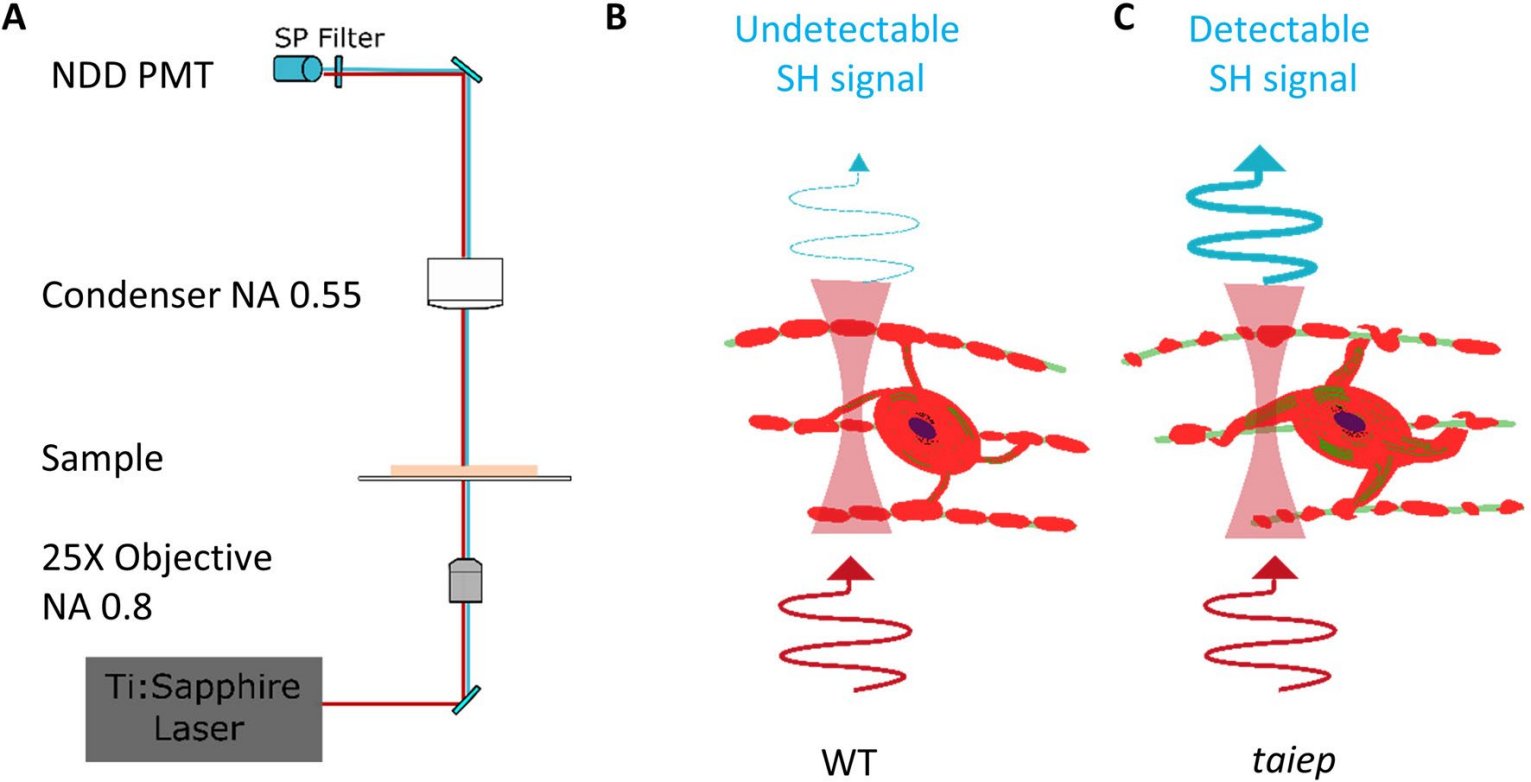
The supernumerary microtubules in mutant oligodendrocytes could emit detectable second harmonic signal. (**A**) Schematic setup of the commercial microscope used for imaging. NDD: non-descanned, PMT: Photomultiplier NA: Numerical aperture. (**B**) The healthy nervous system does not generate detectable second harmonic signal. (**C**) The pathologic white matter of the *taiep* rat emits second harmonic signal.

In this work, the pathological tissue from the CNS of TUBB4A mutant rats was imaged by SHG microscopy (Fig. 1A) with the aim of revealing structural differences due to microtubules amount and organization (Fig. 1B and C). Our hypothesis, that densely packed microtubules in the abnormal/defective oligodendrocytes emit detectable second harmonic signal was tested by comparing SHG images from the white matter to the results obtained from tissue sections immunolabeled and analyzed by fluorescence microscopy.

SHG is a powerful technique capable of revealing the non-linear features of the H-ABC central nervous system, with possible applications to other neurodegenerative diseases and with the potential of complementing the pool of diagnostic techniques used to detect also other myelin and neuronal disorders.

## Results

### SHG imaging of the brain

**Figure 2:**
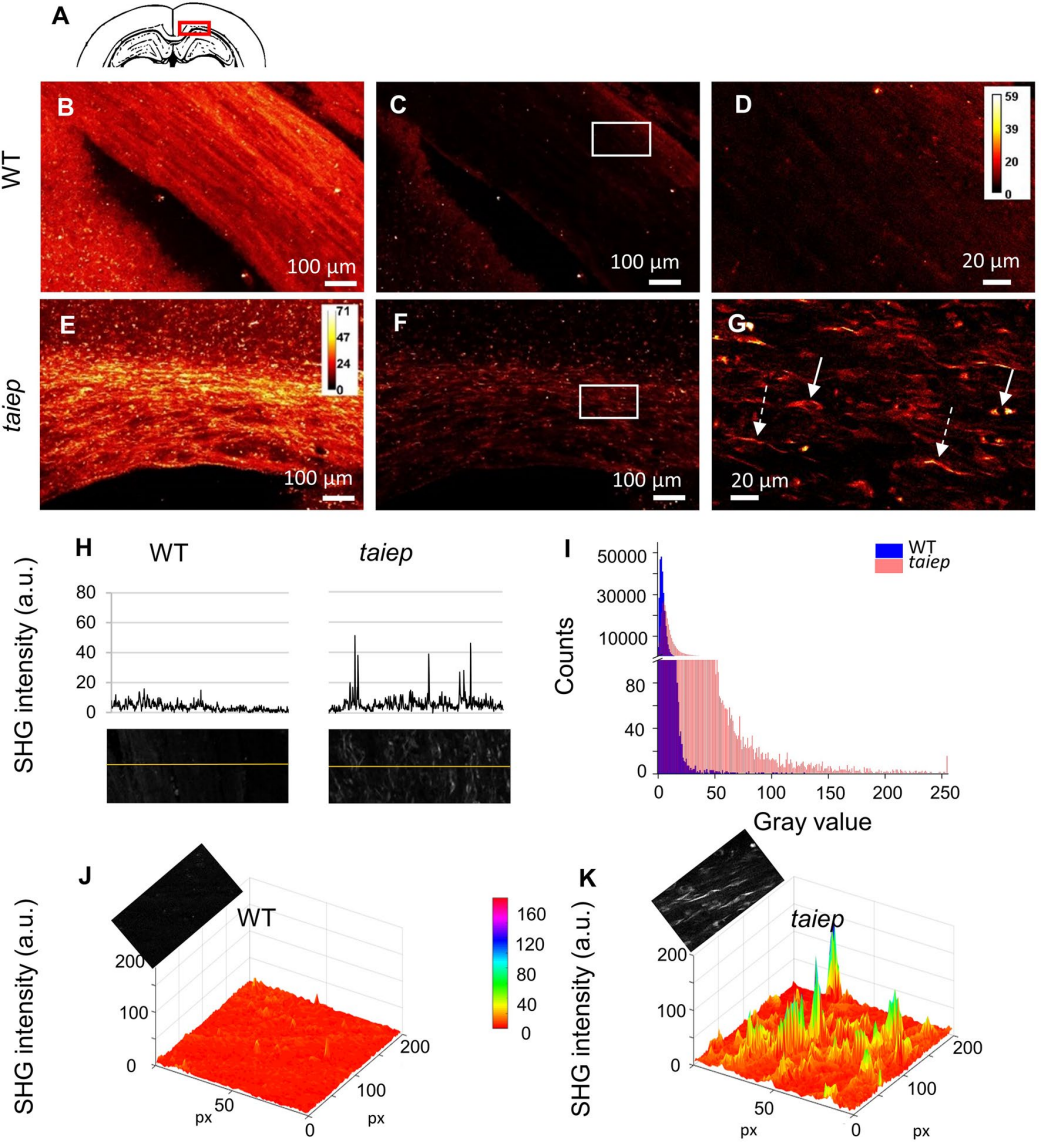
The corpus callosum of *taiep* rats hosts cells that emit second harmonic signals. (**A**) Location of the imaged and analyzed region of the CC. (**B**–**D** and **E–G**) Representative SHG micrographs from the CC of a WT and a TUBB4A mutant rat, respectively; Z-stacks of five images, each spaced 7 μm, were generated. In (**B**) and (**E**) the sum projections of the five planes are shown, to better appreciate the continuity of the emitting structures in the corpus callosum. All animals tested gave identical results. In (**C**) and (**F)** only one plane is shown. Images (**D**) and (**G**) are high magnification images from squares in (**C**) and (**F**), respectively. Arrow, cell bodies; dotted arrow, elongated structures. (**H**) Gray values plotted from the line scans crossing over the fibers in the CC of WT and *taiep* rats; line and image sources are shown just below. **(I**) Gray level histogram obtained from regions of interest (ROIs) in the CC of the rats (26 + 16 images from 3 *taiep* and 2 WT rats). (**J**, **K**) Gray values of surface profiles; intensities obtained from elongated structures in demyelinated regions are higher than those found in similar locations in healthy rats. The calibration bar in (**E**) is valid for (**B,** **C**) and (**F**), and the calibration bar in (**D**) is also valid for (**G**), with brighter colors representing the highest signal intensity. The direction of laser polarization for SHG microscopy coincides with the scale bar orientation. The average power used for SHG imaging was 13 mW.

We first imaged the corpus callosum (CC) (Fig. 2A), the largest white matter (WM) structure of the brain, because it is mainly composed of myelinated axons and therefore more oligodendrocytes can be found there than in other regions. We previously showed the CC progressive demyelination in the animal model and in an H-ABC patient^10^, however, the amount and distribution of CC oligodendrocytes have not been assessed yet.

The images obtained from the CNS of control (wild type, WT) and TUBB4A mutant *taiep* rats showed different patterns of SHG emitting structures. The frequency-doubled, forward-directed signal that arose from the CC of the mutant rats displayed a spatial distribution that resembled elongated cellular processes, cell bodies and also some rounded structures, among the dominating presence of fiber-like structures (Fig. 2E–G). In equivalent regions of control rats, the signal from the CC was always weaker, and no cell body-like distributions were seen (Fig. 2B–D).

The SHG intensity coming from the CC of the mutant rats was higher than that coming from the same organ in control rats, as line plots confirmed (Fig. 2H). By analyzing 26 images from mutant and 16 from healthy rats, we obtained gray values from 26 ROI in the CC (260,000 pixels) for each group of rats. The histogram built with the data depicts the difference in intensity of the images: gray values in WT are below 25 while in *taiep* the majority is below 50 and some pixels reach values up to 250 (Fig. 2I). The surface plot in (Fig. 2K) shows that the pixels with the highest values in the TUBB4a mutant rat were arranged in the shape of elongated structures while in healthy rats the values were much lower and much more homogeneously distributed all over the CC, hindering the recognition of any single thin process (Fig. 2J). We found similar second harmonic signal emitting structures in other tracts like the internal capsule, anterior commissure, and fibers crossing the caudate-putamen of TUBB4A rats (Supplementary information Fig. S1).

### SHG images and immunostaining of the corpus callosum

**Figure 3:**
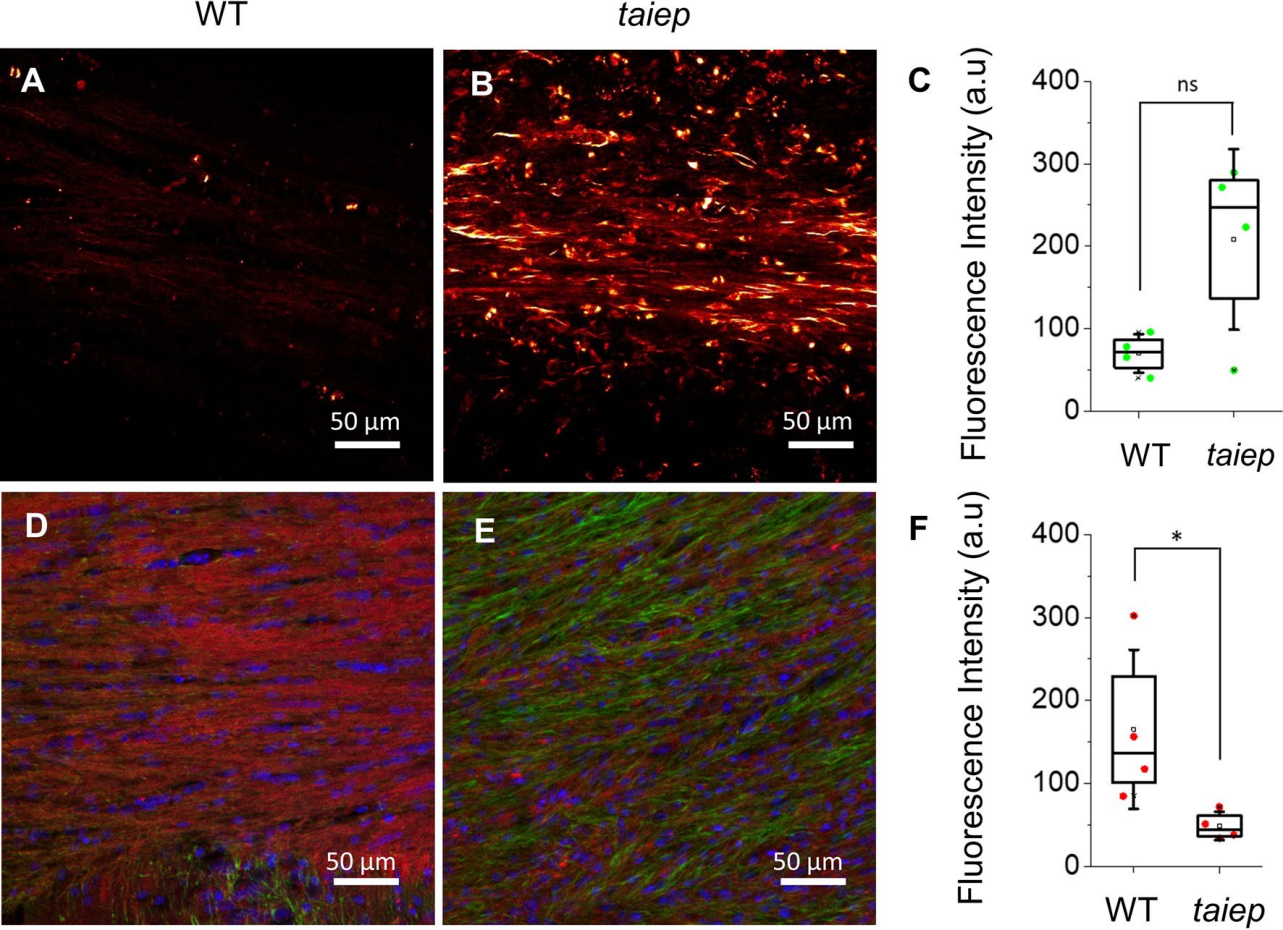
Cells that emit second harmonic signal are located in hypomyelinated regions of *taiep* rats.** (A**, **B**) SHG images from an acute preparation of the CC of a 10 months control and mutant rat, respectively. (**D**, **E**) Confocal images of fluorescently labeled myelin (red), neurofilaments (green), and nuclei (blue) in the CC of WT (**D**) and TUBB4A mutant rat (**E**). The membrane-associated signal appears more intense and organized in control rats than in the mutants and the signal from neurofilaments is less intense in control rats. (**C**) and (**F**) Fluorescence of neurofilaments (green dots) and myelin (red dots), (4 WT and 4 *taiep* rats, *p < 0.05, *p* = 0.03, two-tailed Mann–Whitney test). The direction of laser polarization for SHG microscopy coincides with the scale bar orientation. The average power used for SHG imaging was 13 mW.

To correlate the spatial distribution of SHG signals in healthy and demyelinated WM to the location of neural fibers, the fixed portion of the CNS from the same rats assessed by SHG was immunostained. Neurofilaments (NF) were marked with antibodies and myelin sheets were highlighted with a membrane dye. Major differences were observed in images of neurofilaments in the CC of mutant rats compared to WT. Furthermore, these differences were congruent with the observations obtained with the SHG technique (Fig. 3A and B). The fluorescence emission obtained from stained neurofilaments in white matter tracts was always lower in healthy rats compared to the demyelinated model (Fig. 3E). Conversely, and as expected, myelin staining in the CC of healthy rats was stronger (Fig. 3F). In a qualitative comparison of the images, the neurofilaments bundles in myelinated axons appeared somehow thinner and less intense than bundles in *taiep* rats and the myelin staining displayed the expected distribution in the healthy animal rather than a punctuate pattern in the *taiep* rats (Fig. 3D and E), as previously shown^3^. The graphs were obtained from 4 WT and 4 TUBB4A mutant, 10 months old rats; 2 images from each rat and 2 ROIs in each image of the CC were analyzed (Fig. 3C and F).

### SHG images and immunostaining of the cerebellum

**Figure 4:**
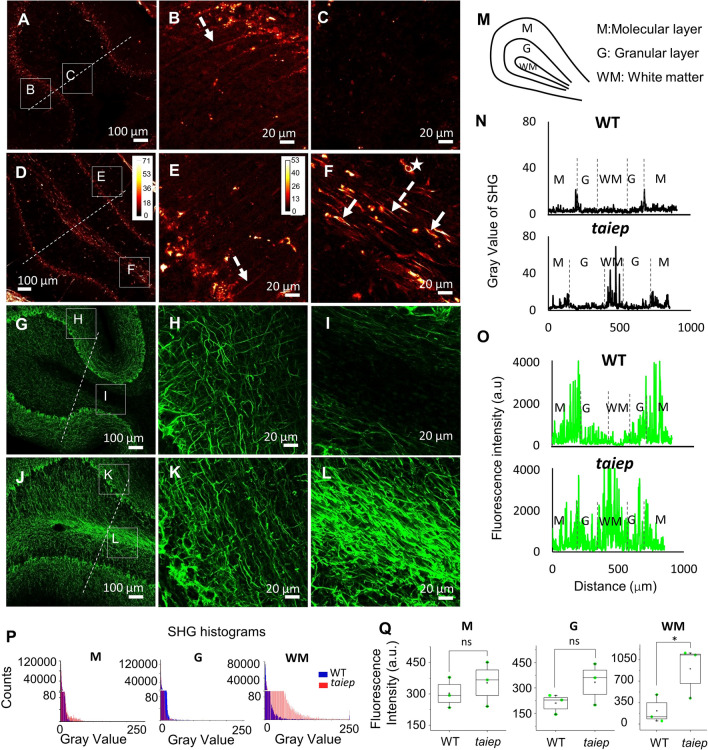
Demyelinated cerebellar white matter in *taiep* rats has second harmonic generating cells and a higher fluorescence intensity from neurofilaments. (**A**–**F**) Representative SHG micrographs from the cerebellum of WT (**A**–**C**) and mutant (**D**–**F**) rats. All animals tested gave identical results. The images are maximum projections from 5 planes separated by 7 μm. (**G**–**L**) Confocal images of stained neurofilaments from the cerebellum of WT (**G**–**I**) and mutant rats (**J**–**L**). (**B, E, H** and **K**) are zoomed regions in the molecular layer and (**C**, **F**, **I,** and **L**), in the cerebellar white matter, where the difference in signal distribution and intensity is better appreciated. In (**F**), arrow, cell bodies; star, intense rounded structure and dotted arrow, fiber-like structure. The calibration bar in panel (**D**), with brighter colors corresponding to the highest signal intensities, is also valid for (**A,** **C** and **F**) while the calibration bar in (**E**) is also valid for (**B**). The direction of laser polarization for SHG microscopy coincides with the scale bar orientation. (**M**) Schematic of cerebellar folia. (**N**, **O**) Representative line graphs of the intensity levels detected along the lines shown in panels **A** and **D** for SHG images, **G** and **J** for NF fluorescence, respectively. In SHG images, the WM of taiep rats shows the highest gray level values of the three layers. The mutant rat also displays higher WM NF fluorescence than the fluorescence from granular and molecular layers (*n* = 3 TUBB4A rats and 3 WT). (**P**) Gray levels histograms obtained from second harmonic signal of the cerebellar layers (2 WT and 3 *taiep* rats). (**Q**) NF fluorescence intensity in the WM of mutant rats was higher than the fluorescence from control rats, no significant difference was found in molecular and granular layers between WT and *taiep* (*n* = 3 *taiep* and 3 WT rats, *p < 0.05, two-tailed Mann–Whitney test). The average power used for SHG imaging was 13 mW.

The hypomyelination and the atrophy of the cerebellum are pathognomonic signs of H-ABC. Together with the profound motor impairment they cause, they are also present in the animal model, therefore we screened the cerebellar white matter for the presence of second harmonic signal emitters. In all TUBB4A mutant rats examined, the SHG active structures in each folium were arranged inside cell bodies or processes and located in the cerebellar WM. The detected signals had a higher intensity than any signal obtained from homologous regions of control animals (Fig. 4A to F). We identified the Purkinje cell layer in the low magnification images of the mutant and control rats' cerebellum. It was also possible to distinguish neural-like structures in the molecular layer (Fig. 4B and E).

To investigate if there was a correspondence between the spatial distribution of SHG and neural fibers in cerebellar layers (Fig. 4M), we assessed the fixed tissue sections by immunofluorescence. We analyzed the fluorescence intensity of the cerebellar layers and the WM (Fig. 4G to L). In control rats the molecular layer displayed the highest fluorescence intensity of neurofilaments (Fig. 4O). Instead in *taiep* rats, the fluorescence signal from fibers in the WM was stronger than fluorescence in the granular and molecular layers (Fig. 4O). Comparing between WT and *taiep* cerebellar regions, only neurofilaments fluorescence intensity from the WM of *taiep* rats was significantly stronger than the fluorescence of WM of control rats (Fig. 4Q).

Signal intensity in SHG micrographs obtained from homologous regions of the cerebellum of the same rats, reproduced the pattern observed in fluorescence images, i.e. dramatic difference in intensity in *taiep* white matter, being the signal from the mutant stronger than that from the WT (Fig. 4A and D), which is also evident from the intensity profile obtained from the folia (Fig. 4N).

In the TUBB4A rat, a strong SHG signal revealed processes and some cell bodies in the WM (Fig. 4D and F). In the molecular layer the nonlinear signal of WT and TUBB4A rats could be associated to axons of granular cells (Fig. 4B and E); those axons, and many more oriented transversally, very likely parallel fibers, were also visible in the NF images (Fig. 4H and K). By analyzing 16 images from mutant and 15 from WT rats, we obtained gray values from 60 ROI in the cerebellar white matter, 30 ROI (300 000 pixels) for each group of rats. The histogram of the white matter (Fig. 4P) illustrates that most gray values of WT are below 25, while in *taiep* most values are below 50, with some pixels displaying values up to 250; for the gray value histograms of the molecular and granular layer, no appreciable difference between taiep and WT is observed.

### Mature oligodendrocytes in white matter

It was shown by transmission electron microscopy (TEM) that oligodendrocytes of *taiep* rats have an unusually high amount of microtubules in the soma and in their processes^22^. In a novel Tubb4a D249N/D249N knock-in mouse model for H-ABC, the engineered mutation affected oligodendrocytes and neuronal survival, with a short life expectancy. A lower number of mature oligodendrocytes was found in the CC of transgenic mice, and this was explained as the effect of inefficient differentiation of oligodendrocyte progenitor cells and cell death. They also found evidence of severe loss of cerebellar granular neurons attributed to alterations of MTs organization in axons^23^.

**Figure 5:**
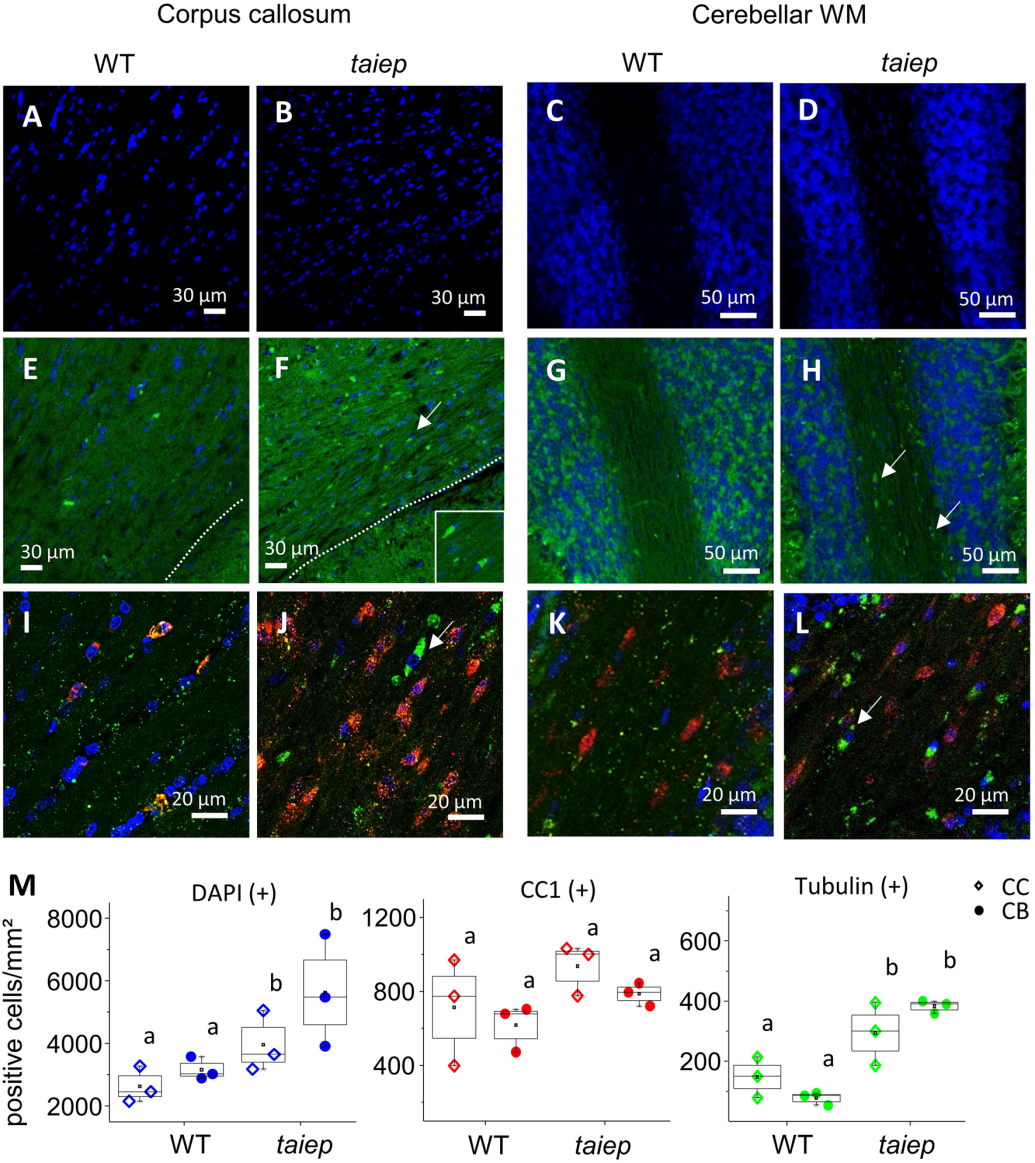
In cerebellar white matter, differences in cellular numbers between *taiep* and WT are more pronounced. (**A**–**D**) More nuclei are observed in the corpus callosum and cerebellar WM of *taiep* rats than in WT. (**E**, **F**) Tubulin staining in the corpus callosum of WT (**E**) and *taiep* rats (**F**); inset in (**F**) shows a magnification of the region pointed by the arrow. (**G,** **H**) Cerebellar WM and granular layer in WT (**G**) and *taiep* rats (**H**), arrows in (**H**) point at cell bodies heavily stained for tubulin. (**I**-**L**) Sections labeled with the mature oligodendrocyte marker CC1 in red, and tubulin in green; corpus callosum of WT (**I**) and *taiep* rats (**J**), cerebellar WM of WT (**K**) and *taiep* rats (**L**). The arrow in (**J**) points to a cell positive for tubulin and the arrow in (**L**) points to a cell positive for tubulin and CC1. (**M**) Quantification of DAPI, tubulin and CC1 positive cells/mm^2^ in the corpus callosum and in the cerebellar white matter. Significant differences were found between genotypes for DAPI (+) cells (F_(1,8)_ = 9.35, *p* = 0.00028) and for tubulin (+) cells (F_(1,8)_ = 37.389, *p* = 0.015), (*n* = 3 WT and 3 *taiep* rats, two-way ANOVA followed by Tukey's multiple comparison test was performed. Different letters indicate significant differences between groups).

To assess oligodendrocytes presence and tubulin overexpression, we immunolabeled the tissues for tubulin and for oligodendrocytes markers, CC1 and Olig2. The nuclear counterstain indicated a higher amount of cells in the CC and the cerebellar WM of *taiep* rats compared to WT (Fig. 5A–D). The tubulin staining was ubiquitous due to the nature of the antibody used, a polyclonal immunoglobulin against alpha/beta-tubulin (Fig. 5E–L), however, in *taiep* rats more cells resulted tubulin-positive and we recognized cell bodies strongly stained for tubulin in the CC and in the cerebellar WM (Fig. 5F–L, arrows). The shape of the structures that were strongly stained for tubulin in *taiep* rat WM sections, closely resembled the cell bodies and rounded structures observed by SHG in the same regions of CC and cerebella of *taiep* rats. Some of the tubulin-positive cell bodies were also positive for the mature oligodendrocyte marker CC1 (Fig. 5J and L) in a similar fashion in *taiep* and WT rats (Fig. 5M), while all the tubulin-positive cell bodies were also Olig2-positive, indicating that the soma-like elements in the CC are oligodendrocytes in any of their developmental stages (Supplementary Fig. 2 A, B, C, D). Oligodendrocytes were found in comparable numbers in adult *taiep* and WT animals (Supplementary Fig. 2 E, F, G), pointing to their misfunction, rather than their loss, as the origin of the myelin defect. As expected, no neurons were found in the CC after staining with the neuronal specific marker NeuN (Supplementary Fig. 2 H, I, J).

**Figure 6:**
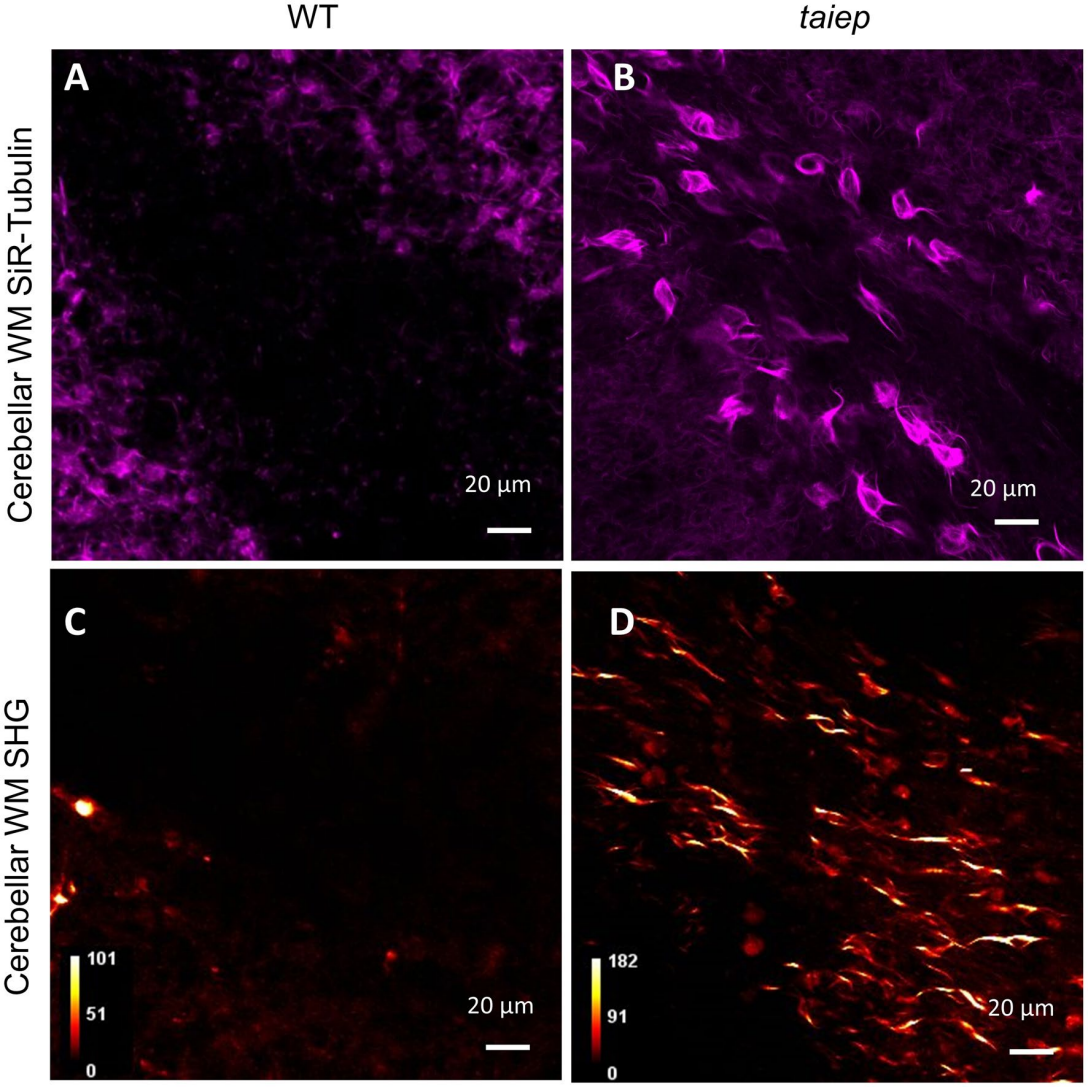
Differences between *taiep* and WT white matter come from microtubules. (**A**, **B**) Acute tissue sections labeled with SiR-Tubulin. In cerebellar white matter of WT rats no cell bodies were observed (A, 2 WT rats aged 2 months). In contrast, cell bodies and processes labeled for tubulin were detected in *taiep* rats WM (B, 2 *taiep* rats aged 8 months). (**C**, **D**) SHG images from white matter of WT and *taiep* rats (2 WT and 2 *taiep* rats aged 6 months). The average power used for SHG imaging was 20 mW.

To verify our findings with an alternative method, we performed live tissue staining using SiR-tubulin and we confirmed that the difference detected by SHG between WT and *taiep* WM is due to tubulin-positive cells, with a morphology closely resembling that which characterizes the positive SHG signal observable in *taiep* (Fig. 6).

## Discussion

In this work, we explored the potential of a nonlinear imaging technique to highlight the changes in the tubulin network caused by a TUBB4A mutation in H-ABC. Our purpose was to image the consequences of the mutation on fresh, unlabeled nervous tissue, by making a direct comparison between the *taiep* model of tubulinopathy and the WT counterpart. We accomplished it by using SHG microscopy to image sections from the CNS of the *taiep* animal model and the images obtained consistently showed remarkable differences between healthy and mutant nervous tissue.

H-ABC is a disease caused by mutations in genes of TUBB4A. In human CNS, TUBB4A is mainly expressed in the cerebellum, followed by the putamen and white matter^24^. Therefore, the microtubules of cells from these areas are the most affected by H-ABC mutations. Importantly, the p.Asp249Asn mutation, the most common among the mutations that cause H-ABC, alters the morphology of oligodendrocytes and possibly of neurons, at least in vitro^1^. The alterations in oligodendrocytes affect the myelin sheath and cause hypomyelination and even myelin degeneration in childhood. The decrease in myelin content, the atrophy of the basal ganglia and the cerebellum, and the thinning of the corpus callosum are the abnormalities observed by MRI in patients. The same MRI features were observed in the *taiep* rat^3,10^, together with the p.Ala302Thr mutation, which has been associated to the myelin defect in the animal model^3,8^. A mutation in the same position of TUBB3 makes microtubules more stable^25^, this suggests that the mutation in *taiep* rats could cause the enhanced stability of microtubules which leads to their observed accumulation^8^.

Microtubules are among the few biomolecules that generate second harmonic signals. SHG is a nonlinear optical phenomenon that occurs when an intense laser pulse interacts with a non-centrosymmetric molecule, causing it to scatter frequency-doubled photons^26^. Recent work indicates that detectable second harmonic signal from microtubules depends not only on the uniform polarization^14^ but also on the number and stability of the microtubules in a bundle^13^. SHG microscopy is therefore sensitive to subtle changes in the organization of the microtubules network. Our hypothesis was that changes in the MTs network, such as the well-known accumulation within oligodendrocytes in TUBB4A mutant rats, could generate changes in the second harmonic signal detected from CNS tissue.

We found that the WM of the H-ABC animal model contains elongated structures and cell bodies that emitted second harmonic signal and that were not observed in similar sections of healthy rats. Moreover, these structures were detected in the corpus callosum and in the cerebellar white matter of mutant rats. Since, (a) microtubules and collagen are the only second harmonic emitters in the CNS ^17^, (b) many tubulin mutations cause microtubule increased stability ^25^, (c) the emitting structures we detected in *taiep* are located mainly in the WM, and (d) the processes imaged by SHG correspond to cell bodies in the WM containing a high amount of tubulin, as seen in SiR-Tubulin labeled slices, the somata-like structures are plausibly oligodendrocytes bodies, while the fibrous structures might be oligodendrocyte processes, abnormally filled with microtubules.

To further understand the differences found between *taiep* and control rats we also analyzed fixed tissue sections of the same animals by fluorescence/confocal microscopy of myelin, neurofilaments and tubulin. As expected, in the corpus callosum of healthy rats, myelin was well organized surrounding the axons, while in TUBB4A mutants there was clear hypomyelination. The fluorescence associated with neurofilaments was consistently higher in the demyelinated axons of the tubulin mutant. This is well depicted in the corpus callosum and the cerebellar folia where intensity from neurofilaments in WM fibers of *taiep* rats was higher than that from the same regions in WT. We hypothesize that in WT animals, tightly packed myelin sheets might interfere with either the excitation/emission of the labeled axonal cytoskeleton or with the proper diffusion of antibodies underneath the insulator; therefore, only a fraction of the total fluorescence could be detected. It is possible that proper myelination could also interfere with excitation and/or SHG emission from microtubules present inside axons and covered by several layers of a birefringent material^21^. Experiments and simulations that replicate the geometry of microtubules inside myelinated axons are needed to prove this hypothesis.

The tubulin staining in the cerebellar white matter of mutant rats showed the presence of cell bodies that were heavily stained for tubulin, while in the wild-type rat, the same staining procedure did not reveal cell bodies. A stronger tubulin fluorescence in cell bodies could indicate a high amount of tubulin, which agrees with our hypothesis that the cell bodies could be those of defective oligodendrocytes of TUBB4A mutant rats. All the cell bodies with high tubulin fluorescence were also positive to pan-oligodendrocyte staining. Oligodendrocytes’ bodies highly immunoreactive for tubulin have already been reported in *taiep* rats in the CC and cerebellar WM^27^, in the anterior medullary velum^28^ and in culture^29^. In previous studies of the mutant rat, electron microscopy has revealed that the body and the processes of the oligodendrocytes have an increased amount of MTs^9,11,22^. In fact, many of the tubulin-positive processes seen in mutant rats could also be related to the cell bodies detected by SHG and tubulin staining, and branch off along the rest of the white matter surrounding them.

Besides the lower second harmonic signal intensity obtained from WM in control rats, we were able to observe microstructural details, like fibers that build up the corpus callosum which were well organized and parallel to each other, as expected. In these same fibers we observed that the second harmonic signal they generated was very homogeneous, as it has been previously reported for the rat corpus callosum^17^. On the other hand, the CC of *taiep* rats imaged by SHG was characterized by fibrillary structures that did not fill homogeneously the entire organ, and by cell bodies with higher second harmonic signal intensity than the surrounding tissue. Those differences can be better appreciated from images generated by summing various planes in *z*, where the CC of WT compared to *taiep* is more homogeneous and fibers are more organized. Cerebellar features of *taiep* rats, imaged by SHG only differ from the controls in the WM. In the molecular layer, we observed neural processes that look very similar in control and mutant rats. Likewise, the signals and appearance of Purkinje cells and cells from the molecular layer are comparable. The differences were observed in the cerebellar white matter where we found cell bodies and elongated structures in the mutant rats, as already discussed. All those differences highlighted by SHG in H-ABC pathological tissue make the technique an exciting option to consider for developing in vivo and ex vivo diagnostic approaches.

The interest in applying label-free techniques comes from the necessity to visualize native tissues to understand the still unknown physiopathology of H-ABC. Usually, the presumptive diagnosis of most neurodegenerative diseases is based on clinical and radiological correlation and definitively corroborated by molecular genetics. The microscopic description of a healthy vs. pathological nervous system tissue, obtained by noninvasive, nonlinear techniques may contribute to the description of the basic cell biology of recently described diseases and with time, it could contribute to diagnostic and therapeutic strategies. Imaging living biological structures without introducing exogenous labels gives the advantage of having access to a molecular conformation that has not been altered by either fixation, permeabilization and/or binding of chemicals or proteins (like antibodies or fluorescent proteins), in contrast to classical, fluorescence-based imaging techniques. Other unique features of nonlinear microscopy include intrinsic optical sectioning, reduction of out of plane phototoxicity, potential compatibility with extended in vivo imaging and, as near-infrared light is used for excitation, deep penetration into tissue^30^. Very few groups have exploited the potential of SHG to study neurodegenerative diseases in animal models. Kwan et al. described how the spectra of intrinsic signals obtained from the pathological lesions of Alzheimer’s disease found in transgenic mice models of Alzheimer’s disease may be useful for its diagnosis^31^. In a recent work, retinal microtubules of the DBA/2 J mouse, an animal model of glaucoma^32^, were analyzed and cytoskeletal deficits were found to predispose axons of the retinal ganglion to atrophy.

Other multiphoton phenomena like third-harmonic generation (THG) or Coherent Anti-Stokes Raman Scattering (CARS) were used to study aspects related to myelination in the central and peripheral nervous system^33–38^. The nonlinear technique used in this work, SHG imaging, has its own advantages regarding the visualization of the CNS. It is molecule-specific, because its signal in CNS only comes from either microtubules or collagen instead of interfaces like in THG. SHG has also been shown to be sensitive to subtle changes in the microtubules network^13^. A technical advantage is that its implementation in brain imaging facilities where two-photon excitation (TPE) microscopy is performed requires simple modifications^39^, therefore is more accessible than THG or CARS microscopy which require completely different laser sources^35,40,41^, besides detection adaptations. Another important advantage is that the laser power typically used for SHG is not expected to produce thermal injury due to harmonic emission (although a certain amount of damage is unavoidable because of two-photon absorption) and it is therefore compatible with prolonged imaging of living cells such as in culture, slices, organoids, fresh biopsies, etc.^13,14,30,31,42–45^.

Specific biomolecules, like NAD(P)H, FAD, retinol, elastin, collagen, and microtubules in the CNS, are known to provide intrinsic emissions when excited with highly energetic short pulses of light^39^. In our study of the CNS, we were able to detect the physiological signal from collagen for example in meninges and choroid plexus (data not shown) and from microtubules in the CC and cerebellum, together with the classical pathological signal coming from the WM tracts in brain and cerebellum of the TUBB4A mutant rats. When considering the contribution of other intrinsic emitters for the detected second harmonic signals, the possibility of NAD(P)H, FAD, and retinol being the source of the intense second harmonic signals in the white matter tracts of the tubulinopathy model is unlikely, since in all the neurodegeneration processes studied so far, the detectable amount of the three molecules decreases after the onset and with the progression of the disease^46,47^. A region of the cerebellum where we detected comparable nonlinear signal in both control and mutant rats was the Purkinje cell layer. This signal has been attributed to TPE from NAD(P)H and FAD in the cytoplasm of Purkinje cells^17^. Although we cannot exclude contamination of the TPE signal in our nonlinear images, it does not affect the differences found between control and *taiep* rats in white matter.

Remarkably, the stabilizing effect of taxol in microtubules, which generates higher intensity second harmonic signals from cell cultures^13^ has also been related to the increase in amount of cytoplasmic microtubules in oligodendrocytes from mouse spinal cord-dorsal root ganglion organotypic cultures^48^ and not in astroglial cells. Moreover, the effect involved microtubules tightly-packed in various orientations in the cytoplasm and also displayed as ordered arrays along endoplasmic reticulum cisternae and nuclear envelope, just as it has been described by TEM in oligodendrocytes of *taiep* rats. In future work, the second harmonic signal intensity from taxol-treated oligodendrocytes crossed with the data about the abundance and stability of microtubules obtained from electron microscopy could help establish a relation between MTs organization and second harmonic signal in oligodendroglial cells.

Further studies on the CNS of the TUBB4A mutant rat at different ages will contribute to the understanding of the relation between the number of cell bodies detected by SHG and the demyelination state. The use of isoform-specific anti-tubulin antibodies will also be determinant in describing how TUBB4A mutations perturb the axonal and the oligodendrocytic cytoskeleton. Finally, this study of a tubulinopathy model using SHG microscopy is the first to expand the applications of the technique for studying neurodegenerative disorders besides those where disruption of axonal MTs has been implicated, like Alzheimer ´s disease and glaucoma, to diseases where a mutation alters MTs organization and number in possibly more than one cell type.

## Materials and methods

### Study design

The study was designed to detect the effects of tubulin mutations in the oligodendrocytes of a tubulinopathy murine model by the innovative application of a microscopy technique based on nonlinear optics. Our work was meant to explore the feasibility of the use of SHG microscopy as a technique able to visualize any possible difference between control and mutant samples, so it was not meant to directly prove any hypothesis of the robustness of the technique for diagnostic purposes (although our results ended up all going in the same direction). The key procedure used to achieve this aim was imaging of acute, unstained tissue section of brain and cerebellum of *taiep* and control rats and it is described in materials and methods.

Due to the lack of previous reports relative to the use of SHG microscopy to visualize excess microtubules in any model of tubulinopathy and also due to the impossibility to estimate in advance any effect size, we have estimated sample size with the resource equation method^46^. With that, our sample size of 14 rats results sufficiently large (in the 10–20 range, assuming 2 groups). We did not exclude any data from the analysis and all the replications gave coherent results. Our experiments were performed in adult animals and to do so we mixed individuals of slightly different ages and from different litters.

Blinding was not performed for the experiments of this work. The reasons include: the student was the only caregiver of the animals previous to their sacrifice. Moreover, the healthy and mutant animals are clearly clinically different at the ages analyzed, which would have required a second person to perform the sacrifice (which was not allowed, for pandemic restrictions) and also, due to the disease, their brains and cerebella are morphologically different, which would have made blinding difficult for the experimenter.

### Tissue preparation and ethics

All procedures described were done in compliance with the Laws and Codes approved in the Seventh title of the Regulations of the General Law of Health regarding Health Research of the Mexican Government (NOM-062-ZOO-1999), and in accordance with the recommendations of the National Institutes of Health Guide for the Care and Use of Experimental Animals and were approved by the institutional committee of bioethics in research of the University of Guanajuato.

*Taiep* myelin mutant rats of the SD strain and healthy controls are raised and maintained in the animal facility of the Institute of Physiology of the Benemérita Universidad Autónoma de Puebla. Animals were genotyped according to the symptoms and not by PCR. Standard conditions are maintained in the animal house, with a temperature of 22 ± 1 °C and a relative humidity of 30 to 45%. The animals are housed in transparent acrylic boxes (60 × 40 × 17 cm) with a bed of sterile wood chips (Aspen, Chip, USA) with free access to a standard diet (Lab Diet 5001, USA, fat = 4.5, protein = 23%, carbohydrates = 34.43%, Kcal/g = 4.07) and purified water. The light–dark cycle is set at 12:12, with the lights on at 07:00. The *taiep* animals used did not present any additional pathology as well as the controls.

Four male taiep and two male Sprague Dawley wild type (WT) 10 months old rats, together with two male taiep and two male WT rats aged 6 months were used for SHG/IHC experiments. Rats were anesthetized with a mixture of ketamine–xylazine (0.125 mg/Kg and 5 mg/Kg, IP), and then sacrificed by decapitation. The brain and cerebellum were removed and the two hemispheres separated. One was used for SHG imaging, obtaining acute coronal brain slices of 160 μm by using a vibratome (Leica VT1200). Tissues were placed in HBSS at 37 °C to prevent microtubules depolymerization during the time between sectioning and SHG imaging. The second hemisphere was immediately immersed in 4% formaldehyde in PBS for immunohistochemical processing. Another group of four taiep and four WT aged 9 months, were perfused with 4% PFA in PBS for additional immunohistochemistry and therefore, not used for SHG imaging.

A summary of the animals used in this work is reported in Supplementary table 1.

### SHG imaging

A LSM-710-NLO confocal microscope (Zeiss) with a Chameleon Vision II titanium sapphire tunable laser (680–1080 nm, 140 fs, Coherent) was used for imaging (Fig. 1 A). Light was focused with a LCI Plan-Neofluar 25X/0.8 immersion objective (Zeiss) and the SHG was collected in the transmission direction (forward path) with a 0.5 N.A. condenser. The excitation wavelength used was 810 nm and non-descanned detection was performed after a low pass filter (LP 490) with photomultiplier tubes (Hamamatsu), using Zeiss NDD units (Fig. 1A). The average power before the objective was 13 mW or 20 mW. The calculated intensities 〈I〉 = 〈P〉/S, considering $\omega_{xy} = \frac{0.325\lambda }{{\sqrt 2 {\text{NA}}^{0.91} }}$^49,50^ are 7.96 MW/cm^2^ and 12.24 MW/cm^2^.

### Fluorescence microscopy

For fluorescence imaging, we used a LSM-710-NLO confocal microscope (Zeiss) equipped with either an LCI Plan-Neofluar 25X/0.8 or an alpha Plan-Apochromat 63x/1.46 Oil Korr M27 immersion objectives.

### Tissue labeling

Fixed tissue blocks were immersed in 30% sucrose in PBS at 4 °C for 24 h. Later, tissue blocks were frozen using tissue freezing medium (ref. 14,020,108,926, Leica) and finally 30–40 μm slices were obtained in a CM 1860 cryostat (Leica).

Sections were marked with anti-APC antibody [CC-1] (ab16794, abcam) and polyclonal anti- Alpha/Beta-tubulin (ATN02, Cytoskeleton), Anti-Neurofilament 200 (N4142, Sigma), anti-NeuN (EPR12763, Abcam) or anti-Olig2 antibody (EPR2673, Abcam), preceded by heat-induced epitope retrieval in sodium cytrate buffer at 95 °C for 10 min, for nuclear epitopes. After washing with PBS, the sections were stained with the Alexa Fluor 555 A21422, Alexa Fluor 488 A11070 and Alexa Fluor 488 A11015 secondary antibodies (Thermo Fisher Scientific). Nuclear staining was performed with DAPI (62,248, Thermo Fisher Scientific). Myelin sheaths were stained with Fluoromyelin red (F34652, Thermo Fisher Scientific) as indicated in the datasheet. Acute tissue sections were incubated at 37 °C in SiR-tubulin (1:100 mL in HBSS, Spirochrome AG CY-SC002) for one hour prior to imaging.

### Analysis and statistics

The open-source FIJI software^51^ was used to convert and reconstruct the images as well as to perform the intensity analysis. The plugin StarDist^52^ was used to count the nuclei. R environment was used for statistical analysis. The box graphs indicate median values with standard error, the symbol “▫” stands for the mean and whiskers represent 5 and 95 percent values. Statistical differences were analyzed with appropriate tests, indicated in figure legends. For all experiments, *p* < 0.05 was considered significant. The data in this study are reported in accordance with ARRIVE guidelines.

## Supplementary Information

Supplementary Information.

## Data Availability

The datasets generated during and/or analysed during the current study are available from the corresponding author on reasonable request.
