# Supplementary material for: H-ABC tubulinopathy revealed by label-free second harmonic generation microscopy
Source: Sci Rep. 2022 Aug 24;12:14417. doi: 10.1038/s41598-022-18370-x (PMC9402540; doi:10.1038/s41598-022-18370-x)
Supplement: Supplementary file 1 — Supplementary Information. [file 41598_2022_18370_MOESM1_ESM.pdf]

H-ABC tubulinopathy revealed by label free second harmonic generation microscopy: supplementary information

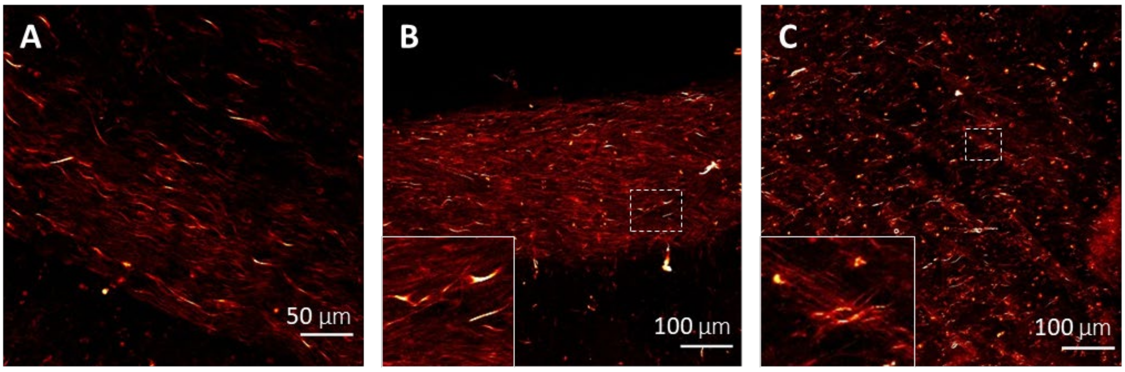

Figure S1. SH signal emitting structures in other tracts of *taiep* rat's brain. (**A**) Processes in the internal capsule. (**B**) Processes in the anterior commissure. (**C**) Fibers crossing the caudate-putamen. Insets show magnification of the dotted regions, with the typical appearance of soma-like structures, detectable throughout the white matter of *taiep* rats.

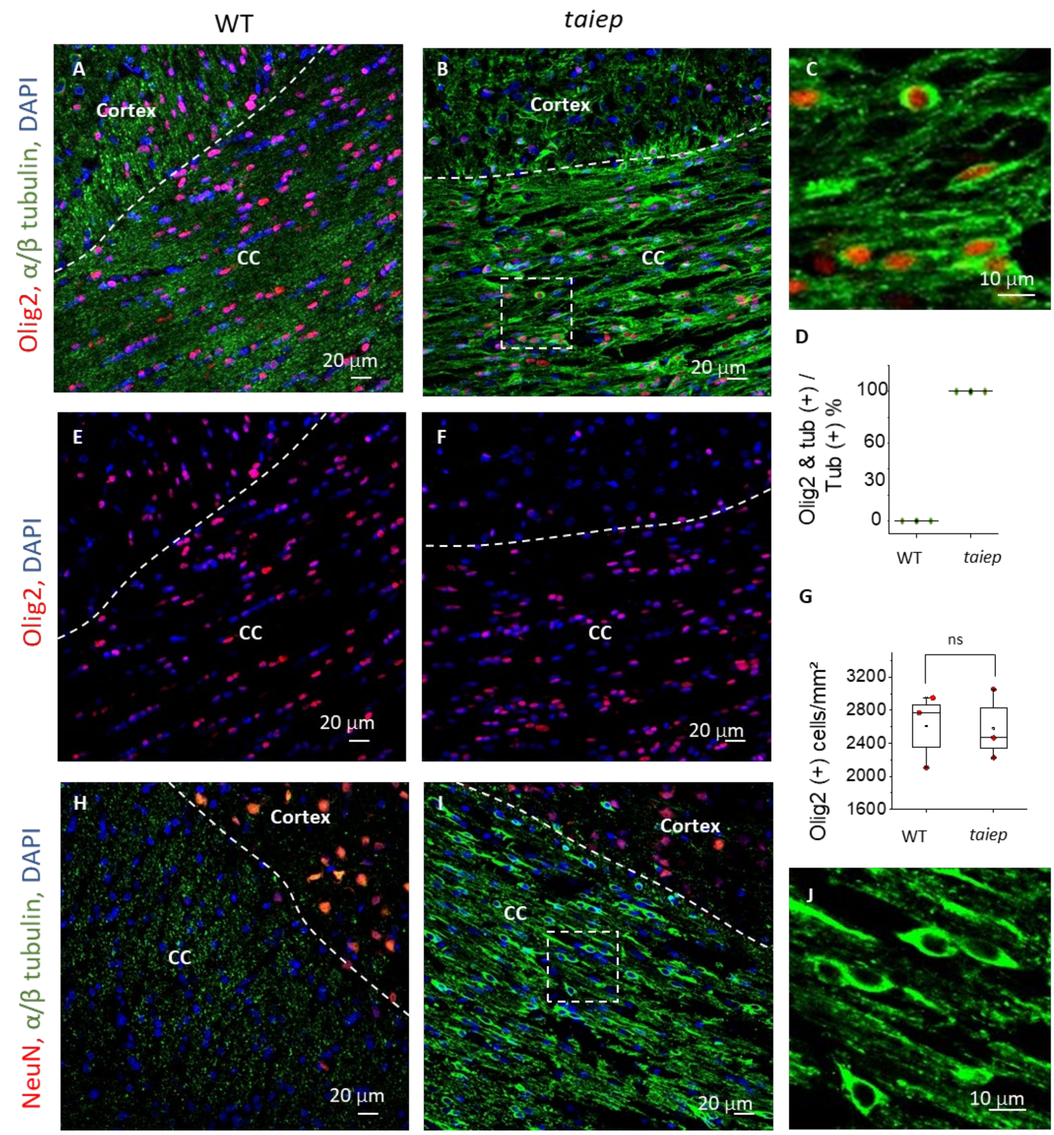

Figure S2. Tubulin positive somata in *taiep* rats CC are positive for the pan-oligodendrocyte marker Olig2 and negative for NeuN neuronal marker. (**A**) Section of adult CC in WT, showing lack of intensely tubulin-stained structures in the white matter. (**B**) Conversely, adult CC of *taiep* rats showed many somata enriched in tubulin, and all of them are also Olig2(+) (**C**, **D**). (**G**) The number of total oligodendrocytes is not significantly different (n=3 *taiep* and 3 WT rats, * $p<0.05$, two-tailed Mann-Whitney) between adult mutant (**F**) and WT (**E**) animals. Tubulin positive cells in *taiep* rats CC do not colocalize with NeuN staining of neurons. Both in WT (**H**) and *taiep* (**I**) CC, NeuN-positive cells are outside the CC, while no soma in the CC displayed NeuN staining (**J**). DAPI nuclear staining was omitted in (**C**) and (**J**) for clarity.

Table S1. Detail of animals used in all the experiments

Experiment	Age	Genotype	Location
**SHG imaging CC and Cb.**	10 months	WT=2<br>*taiep*=4	Figure 2<br>Figure 3<br>Figure 4
**SHG imaging white matter**	10 months	WT=2<br>*taiep*=4	Supplementary figure 1
**Immunohistochemistry Myelin, neurofilaments and nuclei.**	10 months	WT=4<br>*taiep*=4	Figure 3<br>Figure 4
**Immunohistochemistry Tubulin, mature oligodendrocytes, lineage of oligodendrocytes and nuclei.**	9 months	WT=3<br>*taiep*=3	Figure 5<br>Supplementary figure 2
**SHG imaging CC and Cb.**	6 months	WT=2<br>*taiep*=2	Figure 6
**SiR-tubulin**	2 months<br>8 months	WT=2<br>*taiep*=2	Figure 6
